# Supplementary material for: Aion is a bistable anion-conducting channelrhodopsin that provides temporally extended and reversible neuronal silencing
Source: Commun Biol. 2022 Jul 9;5:687. doi: 10.1038/s42003-022-03636-x (PMC9271052; doi:10.1038/s42003-022-03636-x)
Supplement: Supplementary file 2 — Supplementary Information [file 42003_2022_3636_MOESM2_ESM.pdf]

Supplemental Figures

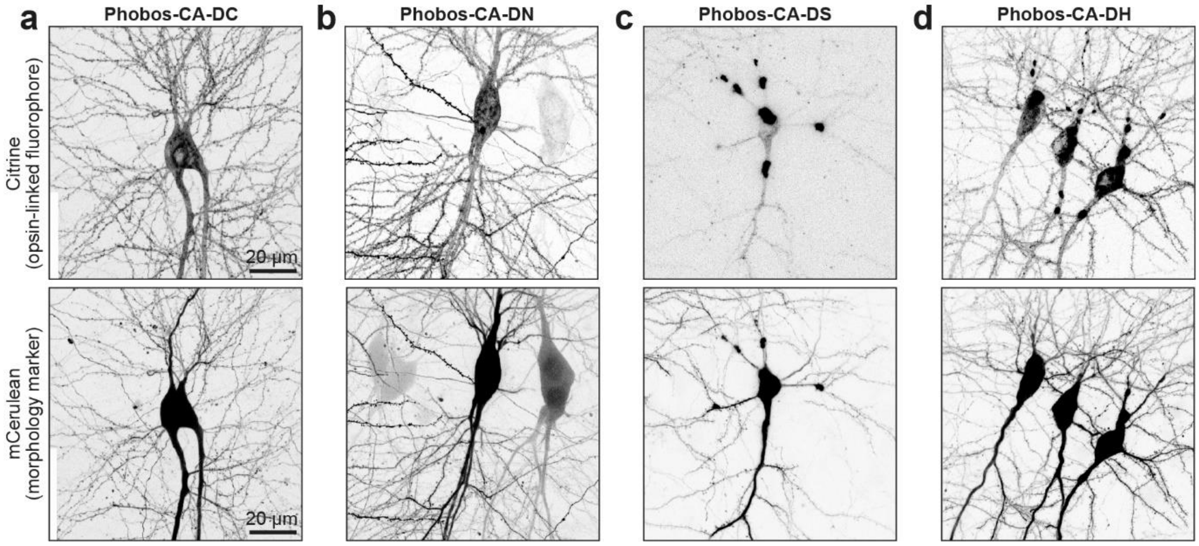

Figure S1. Neuronal expression of Phobos^{CA} D156 mutants. Maximum-intensity projections of two-photon image stacks showing expression of Phobos^{CA}-D156C (**a**), Phobos^{CA}-D156N (**b**), Phobos^{CA}-D156S (**c**), and Phobos^{CA}-D156H (**d**) in CA1 pyramidal neurons after single-cell electroporation in organotypic hippocampal slice cultures. Fluorescence intensity is shown as inverted gray values. Opsin-citrine fluorescence (top row) was mainly localized at the plasma membrane in Phobos^{CA}-D156C (a) and Phobos^{CA}-D156N (b) expressing cells. However, Phobos^{CA}-D156S (c) and Phobos^{CA}-D156H (d) expressing neurons showed poor trafficking and strong protein accumulation in the soma. mCerulean was co-electroporated together with all opsin variants and served as a morphology marker (bottom row).

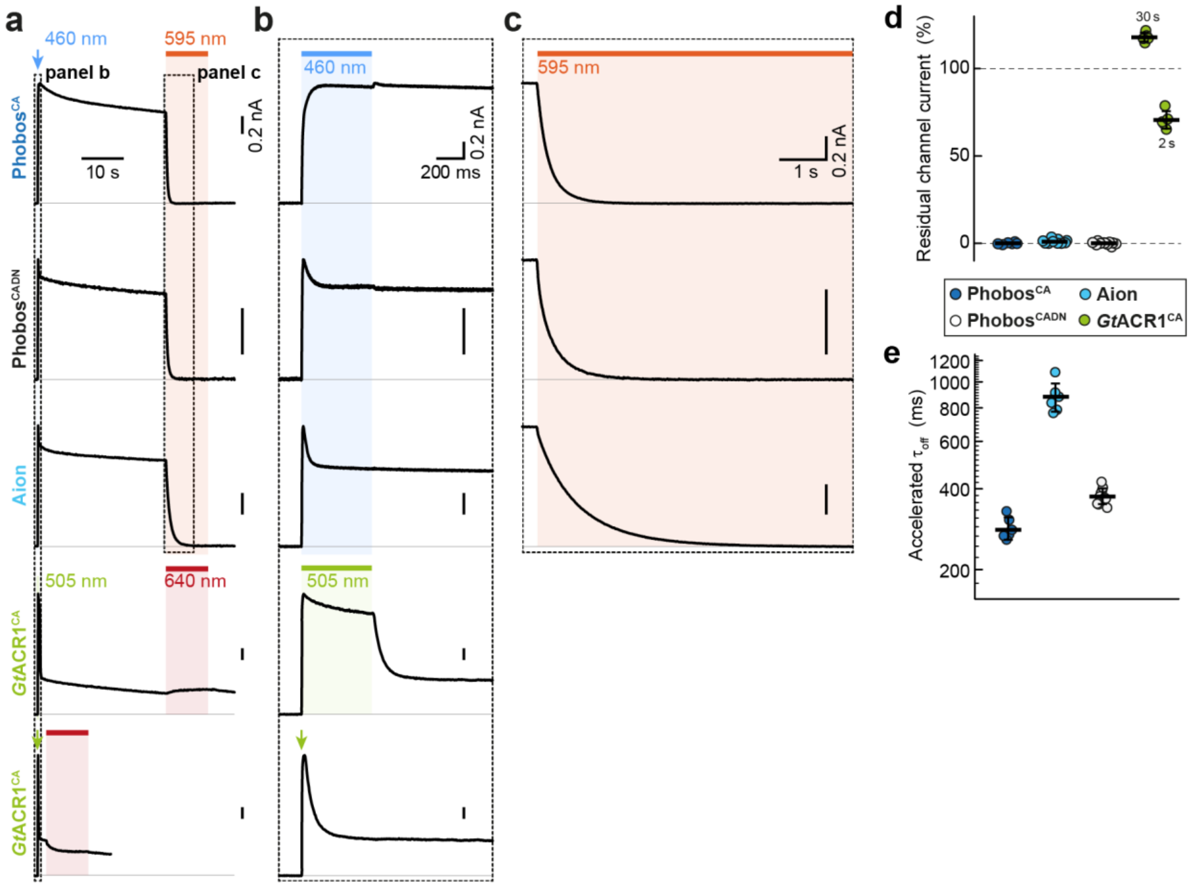

Figure S2. Photoresponse of slow-cycling ACR variants. (**a**) Representative current traces of kinetically modified Phobos^{CA} variants and *Gt*ACR1^{CA}. ACR variants were illuminated with 500 ms (first 4 rows) or 20 ms (last row) light pulses at indicated wavelengths, while termination of photocurrents was accelerated with red-shifted light for 10 s as indicated. (**b**) and (**c**) Temporally expanded views of photocurrent traces from (a) during illumination periods reveal different modes of inactivation (b) and accelerated closing kinetics of Phobos^{CA} variants (c). (**d**) Quantification of the residual photocurrent after orange- or red-light illumination. While *Gt*ACR1^{CA} showed a 30 % photocurrent reduction when red light was applied 2 s post initial activation, application after 30 s caused partial re-opening of the channel. Phobos^{CA} variants could be fully closed by orange light. Black lines correspond to mean values ± SEM, and circles are single measurement data points ($n_{PhobosCA}$ = 6 cells, n_{Aion} = 10 cells, $n_{PhobosCADN}$ = 11 cells, $n_{GtACR1CA}$ = 5 cells). **(e)** Quantification of the accelerated channel closing time constant of Phobos^{CA} variants with orange light ($n_{PhobosCA}$ = 6 cells, n_{Aion} = 7 cells, $n_{PhobosCADN}$ = 11 cells).

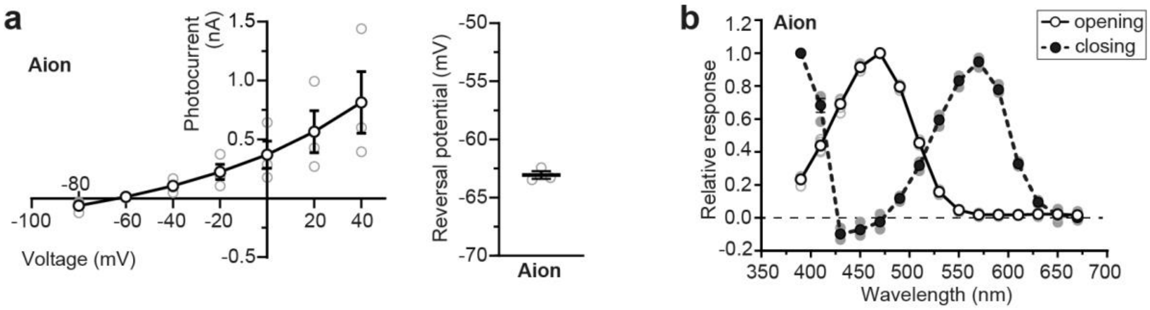

Figure S3. Reversal potential and spectral properties of Aion. (**a**) Quantification of Aion peak photocurrents at different holding potentials (left) and reversal potential (right) in HEK cells during 500 ms illumination with 460 nm at 3.35 mW/mm^2. Black circles and lines show mean ± SEM and single datapoints are shown in grey (n = 3 cells). **(b)** Action spectra of Aion. To determine the activation spectrum (open circles, dashed line) Aion was illuminated with a 10 ms light pulse of different wavelengths in consecutive trials and fully closed with 8 s illumination at 600 nm, between each trial. To determine the inactivation spectrum (filled circles, solid line), Aion was fully opened with a 2 s light pulse at 470 nm and light with different wavelengths was applied for 10 s to accelerate channel closing. Aion was fully closed with 15 s illumination at 600 nm between each trial. Black circles and lines show mean ± SEM and single datapoints are shown in grey ($n_{activation}$ = 5 cells, $n_{inactivation}$ = 3 cells). Note that in some cases SEM was too small to be plotted, see additional source data file for full statistics.

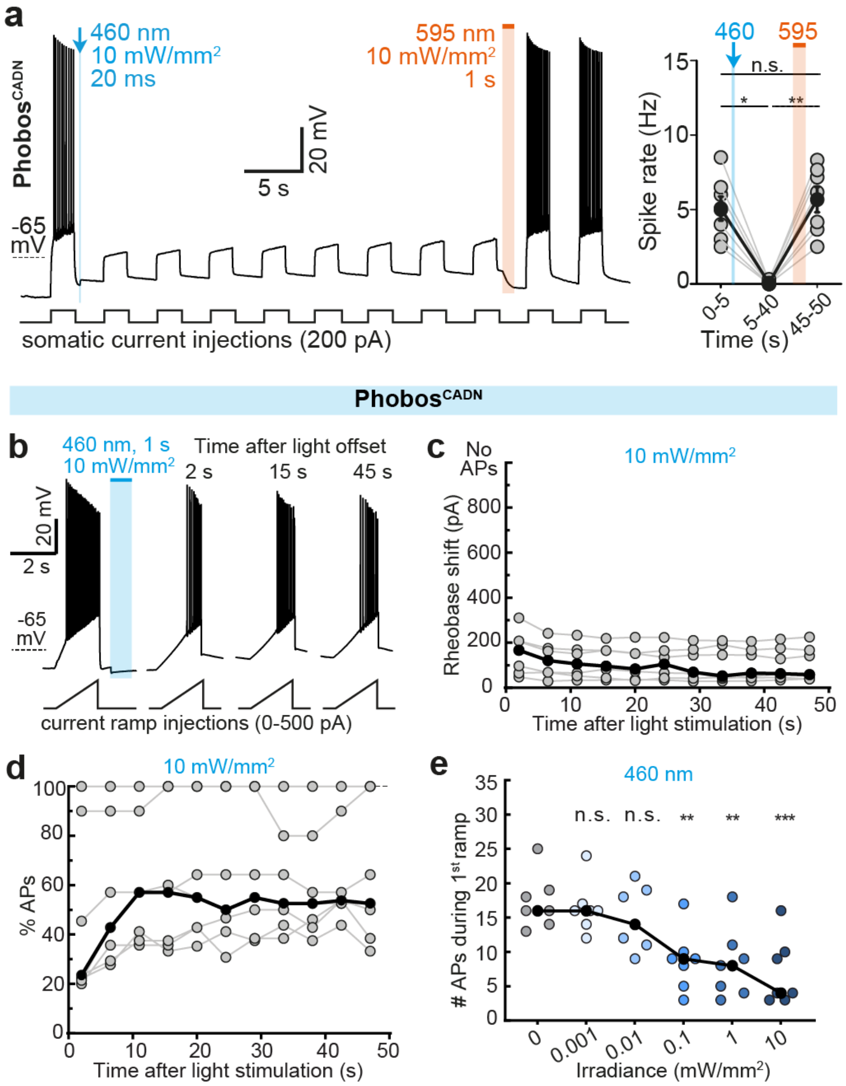

Figure S4. Silencing efficiency of Phobos^{CADN} in CA1 pyramidal neurons. (**a**) Left: Membrane voltage trace showing reversible suppression of depolarization-induced APs by photoswitching Phobos^{CADN} between open and closed state with a blue (460 nm, 20 ms, 10 mW/mm^2) and orange light pulse (595 nm, 1 s, 10 mW/mm^2), respectively. Right: Quantification of spike rate during current injection at indicated time intervals: before channel opening (0-5 s), after channel opening (5-40 s), and after channel closing (45-50 s) in Phobos^{CADN}-expressing CA1 neurons (n = 7 cells). (**b**) Current ramps were injected into Phobos^{CADN}-expressing neurons to induce APs before and after illumination with a short blue light pulse (460 nm, 1 s, irradiances from 0.001 to 10 mW/mm^2). For each ramp, the injected current at the time of the 1st AP was defined as the rheobase. Example membrane voltage traces are shown for the trial in which a light intensity of 10 mW/mm^2 was used. (**c**) Quantification of the rheobase shift and (**d**) the relative change in the number of current ramp-evoked APs during 47 s after light stimulation (460 nm, 1 s, 10 mW/mm^2) (n = 7 cells). (**e**) Number of APs evoked during the first current ramp after opening of Phobos^{CADN} with 1 s blue light at indicated irradiances. Significant AP block was achieved at irradiance values starting at 0.1 mW/mm^2 (n = 7 cells). In all plots, grey (a-d) or colored (e) circles represent single measurement data points and black circles correspond to medians, Friedman test, *$p < 0.05$, **$p < 0.01$, ***$p < 0.001$, n.s. = not significant.

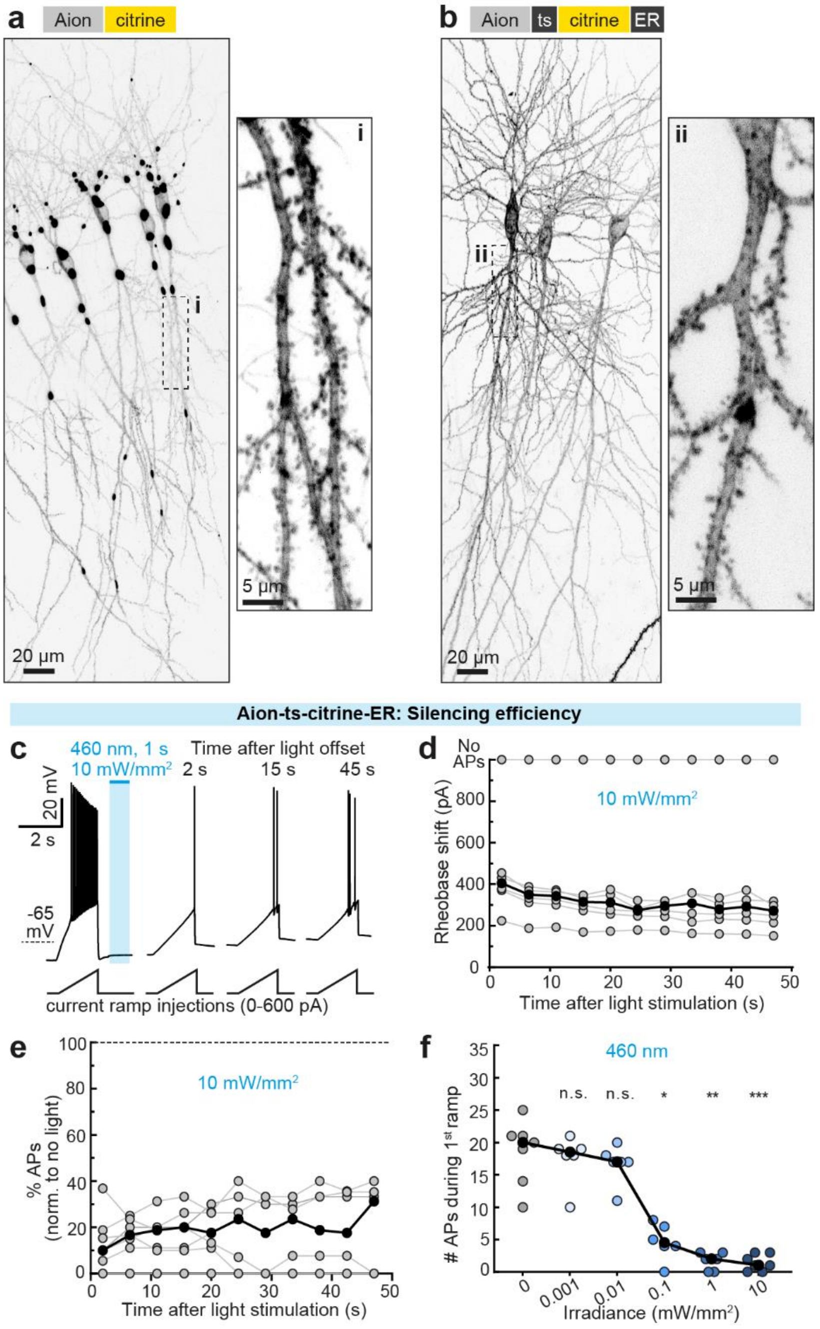

Figure S5. Aion trafficking optimization. (**a**) Maximum-intensity projection of two-photon image stack showing overexpression of Aion-citrine in CA1 pyramidal neurons after single-cell electroporation in organotypic hippocampal slice cultures. In these example cells, opsin accumulations were very prominent in the soma, basal dendrites and in the proximal region of the main apical dendrite. Inset shows magnified view of a portion of the main apical dendrite where main accumulations are not present, revealing efficient membrane localization of non-accumulated Aion. (**b**) Maximum-intensity projection of two-photon image stack of neurons expressing Aion-ts-citrine-ER. In this case, efficient membrane localization with strongly reduced opsin accumulations was observed throughout the cells. Inset shows magnified view of the main apical dendrite. (**c**) Example membrane voltage traces in response to current ramps injected into Aion-ts-citrine-ER-expressing neurons before and after illumination with a 1 s blue light pulse. For each ramp, the injected current at the time of the first action potential was defined as the rheobase. (**d**) Quantification of the rheobase shift and (**e**) the relative change in the number of current ramp-evoked APs during 47 s after light stimulation (460 nm, 1 s, 10 mW/mm^2) (n = 6 cells (d), 7 cells (e)). (**f**) Number of APs evoked during the first current ramp after opening of Aion-ts-citrine-ER with 1 s

blue light at indicated irradiances. Significant AP block was achieved at irradiance values starting at 0.1 mW/mm^2 (n = 7 cells). In all plots, grey (d,e) or colored (f) circles represent single measurement data points and black circles correspond to medians, Friedman test, *$p < 0.05$, **$p < 0.01$, ***$p < 0.001$, n.s. = not significant.

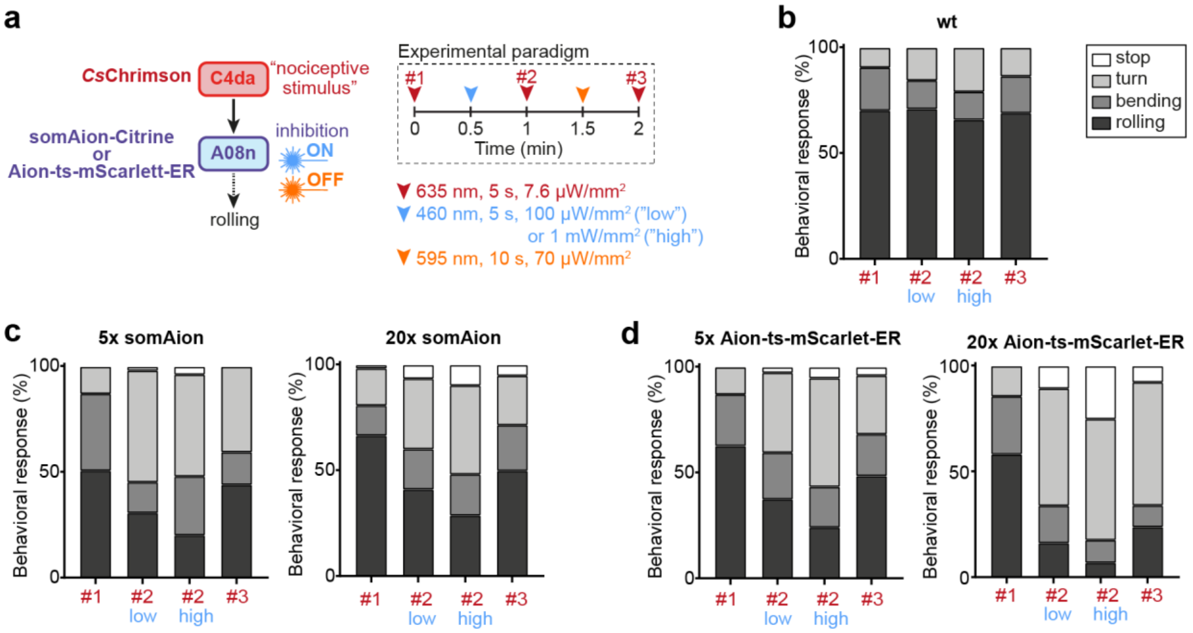

Figure S6. Inhibition of *Drosophila melanogaster* A08n neurons with trafficking-optimized variants of Aion. (a) All-optical paradigm for inhibition of A08n neurons with somAion or Aion-ts-mScarlet-ER and activation of C4da neurons with *Cs*Chrimson in *Drosophila* larvae. **(b-d)** Behavioral responses to *Cs*Chrimson activation in C4da neurons at the indicated time points in wt larvae (b) and transgenic larvae expressing somAion (c, left: 5xUAS, right 20xUAS) or Aion-ts-mScarlet-ER (d, left: 5xUAS, right 20xUAS) in A08n neurons. All Aion variants were able to significantly inhibit nociceptive behavior after a low (100 µW/mm^2) or high intensity (1 mW/mm^2) blue light stimulus, which could be (at least partially) reversed by orange light. For numbers and statistics see supplementary source data file.
