# Supplementary material for: Aion is a bistable anion-conducting channelrhodopsin that provides temporally extended and reversible neuronal silencing
Source: Commun Biol. 2022 Jul 9;5:687. doi: 10.1038/s42003-022-03636-x (PMC9271052; doi:10.1038/s42003-022-03636-x)
Supplement: Supplementary file 3 — Description of Additional Supplementary Files [file 42003_2022_3636_MOESM3_ESM.pdf]

Description of Additional Supplementary Files

File name: Supplementary Data 1
Description: The source data and statistics behind all graphs in the paper.
