# Supplementary material for: Aion is a bistable anion-conducting channelrhodopsin that provides temporally extended and reversible neuronal silencing
Source: Commun Biol. 2022 Jul 9;5:687. doi: 10.1038/s42003-022-03636-x (PMC9271052; doi:10.1038/s42003-022-03636-x)
Supplement: Supplementary file 5 — Reporting Summary [file 42003_2022_3636_MOESM5_ESM.pdf]

nature portfolio

Corresponding author(s): J. Simon Wiegert

Last updated by author(s): Jun 13, 2022

Reporting Summary

Nature Portfolio wishes to improve the reproducibility of the work that we publish. This form provides structure for consistency and transparency in reporting. For further information on Nature Portfolio policies, see our Editorial Policies and the Editorial Policy Checklist.

Statistics

For all statistical analyses, confirm that the following items are present in the figure legend, table legend, main text, or Methods section.

n/a	Confirmed	
☐	☒	The exact sample size (*n*) for each experimental group/condition, given as a discrete number and unit of measurement
☐	☒	A statement on whether measurements were taken from distinct samples or whether the same sample was measured repeatedly
☐	☒	The statistical test(s) used AND whether they are one- or two-sided<br>*Only common tests should be described solely by name; describe more complex techniques in the Methods section.*
☒	☐	A description of all covariates tested
☐	☒	A description of any assumptions or corrections, such as tests of normality and adjustment for multiple comparisons
☐	☒	A full description of the statistical parameters including central tendency (e.g. means) or other basic estimates (e.g. regression coefficient) AND variation (e.g. standard deviation) or associated estimates of uncertainty (e.g. confidence intervals)
☐	☒	For null hypothesis testing, the test statistic (e.g. *F*, *t*, *r*) with confidence intervals, effect sizes, degrees of freedom and *P* value noted<br>*Give P values as exact values whenever suitable.*
☒	☐	For Bayesian analysis, information on the choice of priors and Markov chain Monte Carlo settings
☒	☐	For hierarchical and complex designs, identification of the appropriate level for tests and full reporting of outcomes
☒	☐	Estimates of effect sizes (e.g. Cohen's *d*, Pearson's *r*), indicating how they were calculated

Our web collection on statistics for biologists contains articles on many of the points above.

Software and code

Policy information about availability of computer code

Data collection	Clampex 10.4, Molecular Devices; SutterPatch V2, Sutter Instrument; ScanImage 2017b, Vidrio Technologies; MATLAB 2016b, 2019b, Mathworks; IC Capture, The Imaging Source, Arduino 1.0.6; FIMTrack;
Data analysis	Clampex 10.4, Molecular Devices; SutterPatch V2, Sutter Instrument; MATLAB 2016b, 2019b, Mathworks, ImageJ v1.51t, FIMTrack; Prism 9.0, GraphPad, Excel 2019, Microsoft, Adobe Illustrator

For manuscripts utilizing custom algorithms or software that are central to the research but not yet described in published literature, software must be made available to editors and reviewers. We strongly encourage code deposition in a community repository (e.g. GitHub). See the Nature Portfolio guidelines for submitting code & software for further information.

Data

Policy information about availability of data

All manuscripts must include a data availability statement. This statement should provide the following information, where applicable:

- Accession codes, unique identifiers, or web links for publicly available datasets
- A description of any restrictions on data availability
- For clinical datasets or third party data, please ensure that the statement adheres to our policy

Source data are provided with this paper. All data generated in this study are provided in the Source Data file.

Field-specific reporting

Please select the one below that is the best fit for your research. If you are not sure, read the appropriate sections before making your selection.

☒ Life sciences ☐ Behavioural & social sciences ☐ Ecological, evolutionary & environmental sciences

For a reference copy of the document with all sections, see nature.com/documents/nr-reporting-summary-flat.pdf

Life sciences study design

All studies must disclose on these points even when the disclosure is negative.

Sample size	Sample-size calculations were not performed as the effect size was not known before the study. However, sample sizes for the different experiments presented in this study were matched to published experiments that used similar methodology, model systems and manipulations.<br>HEK cell experiments: DOI: 10.1038/s41467-018-06421-9, DOI: 10.1126/science.1249375, DOI: 10.1038/s41467-021-24759-5<br>Hippocampal slice cultures: DOI: 10.1126/science.1249375, 10.1038/s41467-018-06421-9, DOI: 10.1038/s41598-017-14330-y, DOI: 10.1038/srep14807, DOI: 10.1038/s41467-021-24759-5<br>D. melanogaster experiments: DOI: 10.1038/s41598-017-14330-y, DOI: 10.1038/nn.4580, DOI: 10.1038/s41467-021-24759-5
Data exclusions	In patch-clamp experiments, we excluded recordings that were not stable throughout the duration of the experiment or where a proper recording configuration could not be achieved. HEK-cell recordings with a membrane resistance below 500 MOhm or an access resistance higher than 10 MOhm were excluded as reported in the Methods section. Recordings from hippocampal neurons with a membrane resistance below 50 MOhm and a series resistance over 30 MOhm were not analyzed.
Replication	All experiments were replicated multiple times in biologically independent samples (HEK cells, hippocampal slices, fly larvae). The precise n numbers are reported in the manuscript.
Randomization	In all experiments, the order of light intensities were shuffled. In experiments, where cells were held at different membrane voltages, this parameter was also shuffled. Fluorescent HEK cells were randomly selected for patch-clamp experiments. Hippocampal slice cultures were randomly chosen for single-cell electroporation. Transgene expressing cells were randomly selected for patch-clamp experiments, provided they showed intact morphology in the DIC image. For experiments with D. melanogaster, transgenic animals expressing the transgene of interest were randomly collected from a larger stock.
Blinding	Data analysis of HEK-cell recordings and neuronal recordings was not done blinded, because opsin-mediated effects were apparent in the recordings and thereby revealed the condition. However, pre-established, semi-automatic analysis pipelines were used that did not permit dismissal of any data points or experiments by the analyst based on the analysis result. Analysis of D. melanogaster experiments was done blinded to the transgene expression condition.

Reporting for specific materials, systems and methods

We require information from authors about some types of materials, experimental systems and methods used in many studies. Here, indicate whether each material, system or method listed is relevant to your study. If you are not sure if a list item applies to your research, read the appropriate section before selecting a response.

Materials & experimental systems

n/a	Involved in the study	
☒	☐	Antibodies
☐	☒	Eukaryotic cell lines
☒	☐	Palaeontology and archaeology
☐	☒	Animals and other organisms
☒	☐	Human research participants
☒	☐	Clinical data
☒	☐	Dual use research of concern

Methods

n/a	Involved in the study	
☒	☐	ChIP-seq
☒	☐	Flow cytometry
☒	☐	MRI-based neuroimaging

Eukaryotic cell lines

Policy information about cell lines

Cell line source(s)	HEK-293 cells (ECACC 85120602, Sigma-Aldrich, Munich, Germany)
Authentication	Cell line was authenticated by vendor
Mycoplasma contamination	Tested by vendor and routinely tested by DAPI stain and/or PCR assay

Commonly misidentified lines (See ICLAC register)	n/a

Animals and other organisms

Policy information about studies involving animals; ARRIVE guidelines recommended for reporting animal research

Laboratory animals	Organotypic hippocampal slices were prepared from Wistar rats (Janvier labs) of both sexes at postnatal days 5-7; in vivo experiments were done with transgenic Drosophila melanogaster (A08n-splitGal4, 82E12-Gal4 (2nd chr.), UAS-Kir2.1, 27H06-LexA [BDSC #54751], LexAop-CsChrimson [BDSC #55139], UAS-GtACR1 [BDSC #92983]).
Wild animals	n/a
Field-collected samples	n/a
Ethics oversight	All procedures were performed in compliance with German law according and the guidelines of Directive 2010/63/EU. Protocols were approved by the Behörde für Gesundheit und Verbraucherschutz of the City of Hamburg.

Note that full information on the approval of the study protocol must also be provided in the manuscript.
